# Gamma-Irradiated Fowl Cholera Mucosal Vaccine: Potential Vaccine Candidate for Safe and Effective Immunization of Chicken Against Fowl Cholera

**DOI:** 10.3389/fimmu.2021.768820

**Published:** 2021-11-30

**Authors:** Bereket Dessalegn, Molalegne Bitew, Destaw Asfaw, Esraa Khojaly, Saddam Mohammed Ibrahim, Takele Abayneh, Esayas Gelaye, Hermann Unger, Viskam Wijewardana

**Affiliations:** ^1^ College of Veterinary Medicine and Animal Science, University of Gondar, Gondar, Ethiopia; ^2^ Health Biotechnology Directorate, Ethiopian Biotechnology Institute, Addis Ababa, Ethiopia; ^3^ MSc Program on Vaccine Production and Quality Control, Pan Africa University for Life and Earth Sciences Institute (PAULESI), University of Ibadan, Ibadan, Nigeria; ^4^ Vaccine Research and Development Directorate, National Veterinary Institute, Debre Zeit, Ethiopia; ^5^ Animal Production and Health Section, Joint Food and Agriculture Organization (FAO)/International Atomic Energy Agency (IAEA) Centre of Nuclear Techniques in Food and Agriculture, International Atomic Energy Agency (IAEA), Vienna, Austria

**Keywords:** chicken, fowl cholera, gamma radiation, mucosal vaccine, *P. multocida*

## Abstract

Fowl cholera (FC) caused by *Pasteurella multocida* is among the serious infectious diseases of poultry. Currently, formalin inactivated FC (FI-FC) vaccine is widely used in Ethiopia. However, reports of the disease complaint remain higher despite the use of the vaccine. The aim of this study was to develop and evaluate gamma-irradiated mucosal FC vaccines that can be used nationally. In a vaccination-challenge experiment, the performance of gamma-irradiated *P. multocida* (at 1 kGy) formulated with Montanide gel/01 PR adjuvant was evaluated at different dose rates (0.5 and 0.3 ml) and routes (intranasal, intraocular, and oral), in comparison with FI-FC vaccine in chicken. Chickens received three doses of the candidate vaccine at 3-week intervals. Sera, and trachea and crop lavage were collected to assess the antibody levels using indirect and sandwich ELISAs, respectively. Challenge exposure was conducted by inoculation at 3.5×10^9^ CFU/ml of *P. multocida* biotype A intranasally 2 weeks after the last immunization. Repeated measures ANOVA test and Kaplan Meier curve analysis were used to examine for statistical significance of antibody titers and survival analysis, respectively. Sera IgG and secretory IgA titers were significantly raised after second immunization (*p*=0.0001). Chicken survival analysis showed that intranasal and intraocular administration of the candidate vaccine at the dose of 0.3 ml resulted in 100% protection as compared to intramuscular injection of FI-FC vaccine, which conferred 85% protection (*p*=0.002). In conclusion, the results of this study showed that gamma-irradiated FC mucosal vaccine is safe and protective, indicating its potential use for immunization of chicken against FC.

## Introduction

Poultry production contributes significantly to the livelihoods of farmers and to the national economic system. However, it is hampered by various factors, including poor husbandry practices and poultry diseases (1). Fowl cholera (FC), which is caused by *P. multocida*, is among the serious infectious diseases of poultry. The disease is present globally and endemic to most parts of Ethiopia with significant economic losses associated with reduced production and mortality (2). It is vital and preferable to develop vaccines from locally circulating strains to provide a robust protection (3). Despite the contribution by the locally produced formalin-killed FC vaccine in reducing the disease burden, FC remains to be a big challenge to the poultry sector in the country. This in part can be explained by the fact that the current used formalin-inactivated FC vaccine produced by the National Veterinary Institute (NVI) confers a short duration of protection (4). In addition, owing to its nature of being inactivated parenteral preparation, the vaccine is expected to be a poor inducer of mucosal immunity, which is the desired protective immunity against mucosal pathogens such as avian *Pasteurella* (5).

The route of vaccine administration plays an important and significant role in practical usage. The fact that parenteral vaccines induce little-to-none mucosal immunity makes them poor and ineffective choice to immunize against mucosal pathogens (6). In addition, mucosal vaccines are easy to administer and is preferable in case of vaccination campaigns and in farm settings where there is large number of chickens to be vaccinated (7). In general, parenteral preparations induce short-lived humoral immunity, which necessitates booster doses. However, mucosal vaccines elicit long-lived immunity of both humoral and cellular nature (8). Therefore, there is an urgent need to develop effective and safe mucosal vaccines that confer long duration of protection against mucosal pathogens.

The commonly used chemical inactivation methods has limitations associated with safety (probably correlated with the high endotoxin level) and efficacy due to modification of antigenic components of bacteria, making it less immunogenic and potent. Furthermore, these vaccine antigens are mostly presented through major histocompatibility complex (MHC)-II but not MHC-I pathways by antigen-presenting cells and do not result in an efficient cell-mediated immune response that is crucial against many pathogens. Considering recombinant method of vaccine development against FC might not be a feasible approach for developing countries because they need high technical expertise, high-technical facilities, and resource limitations, as well as the immunity conferred is very limited and narrow (9).

Radiation inactivation of pathogens has potential applications in sterilization and the manufacture of biological reagents and laboratory supplies (10). Exposure to optimum doses of gamma radiation disrupts the genetic material of the pathogens, making the microorganism unable to replicate, so it cannot establish an infection yet leaving some residual metabolic activity. Therefore, the irradiated microorganisms may still find its natural target in the host and could effectively be immunogenic (11). The major advantage of ionizing radiation in vaccine development compared to ultraviolet or chemical agents is its ability to effectively penetrate through most biological materials and specifically target nucleic acids whilst causing less damage to surface antigenic protein, making it preferable to develop safe and simple vaccines (12). Gamma-irradiated vaccines appear to be more effective than formalin-killed vaccines against disease and have the added advantage of a longer storage life than live vaccines (13). Therefore, the present study was aimed to develop an improved gamma-irradiated inactivated vaccine against fowl cholera that stimulates the enhanced mucosal immune response and is easy for application at the rural setting.

## Materials and Methods

### Study Site

The study was conducted at the National Veterinary Institute (NVI), Bishoftu; National Institute for Control and Eradication of Tsetse Fly and Trypanosomosis (NICETT), Addis Ababa; and Ethiopian Biotechnology Institute, Addis Ababa, from November 2020 to June 2021.

### Experimental Chicken and Their Management

In this experiment, 250 3-week-old specific antibody negative (SAN) against FC Bovans brown chickens were used. The parent stock was not vaccinated against FC. Chicks used for all the experiments were raised under intensive management system. The animal experiment rooms were cleaned with disinfectants and fumigated with formalin before the introduction of chicks and bedded with disinfected wood shavings. The chickens had access to feed and water *ad libitum* throughout the experiment.

### Preparation of Avian *P. multocida* Inoculum: For Preliminary Study, Vaccine Preparation, and the Challenge Study

Working seeds of Avian *P. multocida* biotype A (MK802880, NVI) were used for vaccine preparation. Lyophilized *P. multocida* biotype A was diluted with 2 ml of tryptose soya broth (TSB), homogenized well and then inoculated into sterile tryptose soya agar (TSA) supplemented with 10% horse serum and incubated at 37°C overnight. The identity of this isolate was confirmed by both phenotypic and molecular standard tests. A single colony was transferred to 2 ml tube containing TSB with 10% horse serum and incubated for 7 h at 37°C. Then 0.5 ml of the broth culture was transferred into 30 ml TSB supplemented with 10% horse serum and incubated overnight. The purity of the *P. multocida* type A (PA) inoculum was checked and inoculated into PA production media at the ratio of 7 ml of inoculum, 7 ml of glucose, and 3 ml of serum per 300 ml of *P. multocida* production media, then incubated for 24 h with slow agitation at 80 rpm (14). The culture was harvested at the pH of 5.5 to 6.2, which is known to correspond to the desired titer of 10^9^ CFU/ml and above as determined by the plate count method. In addition, avian *P. multocida* strain was used as challenge strains in the test of the vaccines. Freeze-dried stock was reconstituted with 2 ml tryptose broth (TB), and suspensions were streaked on tryptose soya agar (TSA) plates incubated for 24 h at 37°C. The culture was checked for purity and identity. From the culture on TSA, a typical colony was inoculated to 200 ml TSB and incubated for 7 h at 37°C. These cultures were then adjusted spectrophotometrically at 450 nm (0.475 OD value) and serially diluted in TSB to obtain the desired titer for challenge (3.5×10^9^ CFU/ml).

### Determination of Appropriate Gamma Radiation Dose for Optimum Inactivation of Avian *P. multocida*

The PA production media containing culture was centrifuged at 4,000×g per minute at 4°C for 20 min after determining the time required to obtain the desired titer (5.6×10^9^ CFU/ml). The supernatant was discarded, and the cell pellet was washed twice with PBS and resuspended in PBS with equal volume in falcon tubes and subjected to gamma irradiation with doses ranging from 0.5 to 3 kGy at a dose rate of 1.56 kGy/h using a cobalt 60 irradiation machine (MDS NORDION, Canada) (15, 16).

The falcon tubes containing the culture were placed vertically and securely in the gamma chamber and irradiated for different time periods according to the required doses of gamma rays. The temperature inside the gamma chamber was maintained at 37–40°C. After completion of irradiation, each tube was carefully removed from the gamma chamber and immediately stored at −4°C for further use. Non-irradiated controls underwent the same procedure except irradiation. The facility at the National Institute for Control and Eradication of Tsetse Fly and Trypanosomosis (NICETT) at Addis Ababa was utilized for this purpose. Bactericidal activity of the radiation dose was assessed by subculturing of serial dilution of *P. multocida* cells plated on tryptose soya agar plates to quantify CFU. Various irradiation doses were examined to find the lowest optimum irradiation at the margin of the lethal dose (17).

### Safety and Immunogenicity Study of Avian *P. multocida* Irradiated at Different Dose of Gamma Radiation

Avian *P. multocida* preparations irradiated at four consecutive irradiation doses close to complete lethal dose and adjuvanted with 20% of Montanide/01 PR gel adjuvant were evaluated for their immunogenicity and safety. The inoculum preparation of avian *P. multocida* used for challenge was done as indicated in above. Thirty chickens were randomly divided into five groups with six chickens in each group and were intranasally inoculated with 1 ml of candidate mucosal FC vaccine irradiated with 0.9 kGy (group 1), candidate mucosal FC vaccine irradiated with 1 kGy (group 2), candidate mucosal FC vaccine irradiated with 1.1 kGy (group 3), candidate mucosal FC vaccine irradiated with 1.2 kGy (group 4), and control inoculated with PBS (group 5). Following vaccination, chickens were monitored daily for any behavioral changes. Blood samples were collected from the wing vein at days 0, 14, and 21 post-vaccination to determine the antibody titer raised against avian *P. multocida* biotype A (PA) using the indirect ELISA (Product code: PMS-CHICK-5P, IDvet, France). Safety was assessed by monitoring administration site reactions such as pain and swelling, systemic reactions like fever and anorexia, and lesion in the liver and spleen.

### Formulation of the Candidate Gamma-Irradiated Mucosal FC Vaccine

The 1 kGy gamma-irradiated avian *Pasteurella multocida* was chosen for the vaccine preparation since it performed best in antibody production using I-ELISA test. The inoculum preparation of avian *P. multocida* was done as mentioned in section 3.2.1. The irradiated culture of avian *P. multocida* (5.6×10^9^CFU/ml) was adjuvanted with Montanide/01 PR gel to form the final vaccine preparation. The proportion of Montanide/01 PR gel adjuvant is made to comprise 20% of the antigen preparation as recommended by the adjuvant manufacturer (18). Then, the vaccine was dispensed into vials of 50 ml volume capacity and checked for its purity and sterility by using Gram’s stain and culturing on sterility test media such as tryptose agar, tryptose broth, and Sabouraud agar media. Finally, the gamma-irradiated fowl cholera vaccine was found free from any contamination.

### Evaluation of the Final Candidate Vaccine

**Figure 1:**
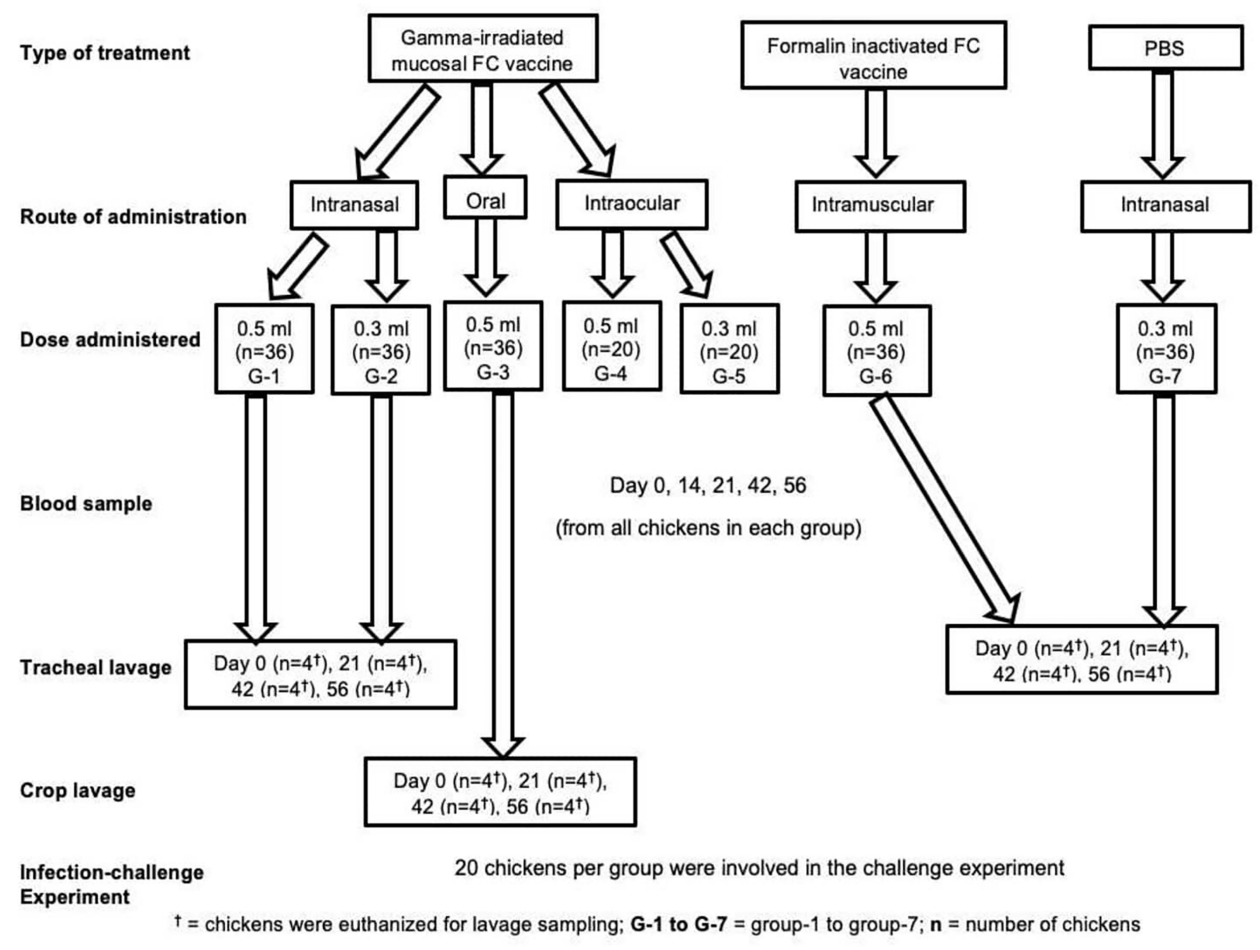
Experimental design groups; treatment, comparator, and control, route of vaccine administration, and number of chickens in each group, number of chickens used in sampling, and number of chickens used in efficacy study.

Chickens were allocated into seven groups, G-1 to G-5 based on the dose of candidate mucosal FC vaccine they received and the route of administration. Thirty-six (36) chickens from both G-1 and G-2 received the vaccine intranasally (IN) at a dose of 0.5 and 0.3 ml, respectively. Similarly, 36 chickens from G-3 received the vaccine at a dose of 0.5 ml orally. On the other hand, 20 chickens both from G-4 and G-5 were administered with 0.5 and 0.3 ml of the vaccine intraocularly (IO), respectively. All the chickens (G-1 to G-5) received three doses of the vaccine preparations at 3-week interval. Another two groups of chickens, G-6 (36 chickens) and G-7 (36 chickens), were used as a comparator and placebo control, respectively. Chickens in G-6 were administered three doses of 0.5 ml of the commercial formalin-inactivated FC vaccine (1/20) at 3-week interval intramuscularly. Finally, all chickens from G-7 received PBS (**Figure 1**).

### Assessment of the Safety of Mucosal FC Vaccine

Evaluation of the safety of the vaccine was done according to the OIE manual for vaccine safety parameter (3), vaccinated chickens were observed the whole day starting from the time of vaccination up to the end of the experiment on a daily basis, and any deviation from normal health using observation of vital signs was recorded: depression, anorexia, ruffled feather, and any reaction at the site of injection.

### Assessment of Serum and Mucosal Antibody Response

Antibody responses, serum IgG, and secretory IgA in chickens were determined by ELISA. Blood samples were collected on days 0, 14, 21, 42, and 56 of the experiments. In addition, four chickens in each group except intraocular route were sampled and euthanized on days 0, 21, 42, and 56 of the experiments, and the tracheal and crop lavage was performed (**Figure 1**). Then, the sera or tracheal and crop lavage solutions were subjected to ELISA procedures. Antibody responses in the chicken sera were determined by measuring of the IgG titers using a commercial indirect ELISA test kit (Product code: PMS-CHICK-5P, IDvet, France). In addition, the secretory IgA was measured using a chicken IgA sandwich ELISA Kit (CAT. No: MBS564152MyBioSource, San Diego, USA). The average antibody titer and the standard error of the mean (SEM) of each group were computed according to the company’s recommendation.

### Assessment of Efficacy of the Candidate Vaccine

As indicated in **Figure 1**, 20 chickens from all groups were challenged with avian *P. multocida* at a dose of 3.5×10^9^ CFU/ml 2 weeks after the final vaccination. Preparation of avian *P. multocida* for challenge study was done as indicated in the above section. The chickens were followed up for clinical signs and mortality for 14 days. Necropsy and bacterial isolation were conducted on dead chickens. The gross lesions were recorded, and lungs, livers, and spleens were collected for bacterial isolation by direct culture using TSA with 10% horse serum followed by identification through morphology, staining, culture, and finally by species-specific PCR.

### Data Analysis

GraphPad Prism 9 was used to perform statistical analysis. The antibody titers between the vaccinated groups and the non-vaccinated control group were performed using a repeated measures ANOVA test and Tukey multiple comparisons. The level of significance was recorded at p<0.05. The data were presented as individual values for each experimental group. Mean and standard error of means are indicated in lines and error bars. The survival of chickens was compared between different treatment and *in vivo* infection challenge groups using Kaplan-Meier curve analysis.

## Results

### Effects of Gamma Irradiation on Avian *P. multocida*

**Figure 2:**
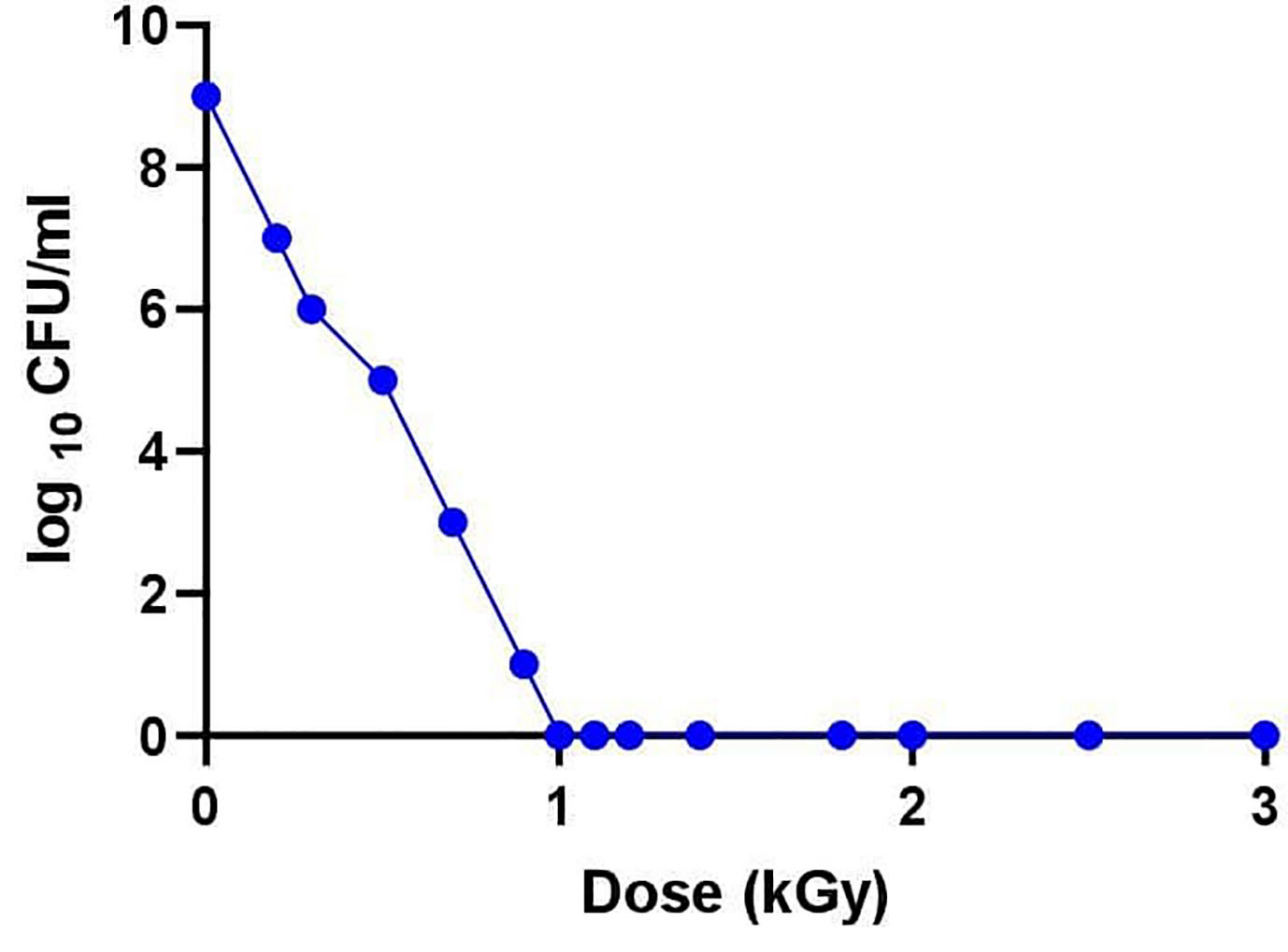
After determination of the desired titer (5.6×109 CFU/ml), the broth culture was centrifuged at 4,000 revolutions per minute at 4°C for 20 min. The supernatant was discarded, and the pellet of cells was washed twice with PBS and resuspended in 5 ml PBS in 15 ml falcon tubes and subjected to gamma irradiation with doses ranging from 0 to 3 kGy at a dose rate of 1.56 kGy/h using a cobalt 60 irradiation machine (MDS NORDION, Canada). Bactericidal activity of the radiation dose was assessed by subcultivation of serial dilution of *P. multocida* cells plated on tryptose soya agar plates to quantify CFU. Various irradiation doses were examined to find the lowest optimum irradiation at the margin of the lethal dose.

Irradiation experiments were conducted to determine the dose required for the inactivation of avian *P. multocida*. An exponential decrease in viability of avian *P. multocida* was observed while increasing the dose of gamma irradiation. After 48 h of culturing, avian *P. multocida* exposed to doses more than or equivalent to 1 kGy of irradiation had fully inhibited replication, and no growth was seen (**Figure 2**). The susceptibility of surface structural proteins to reactive oxygen species (ROS) damage increases when the radiation dose is higher than the level that completely abolished avian *P. multocida*. Therefore, the lethal dose (1 kGy) and three doses close to the lethal dose (0.9, 1.1, 1.2 kGy) were used to select immunogenic dose and safety of irradiated avian *P. multocida* in chicken.

### Immunogenicity and Safety of Radiation Inactivated Avian *P. multocida* Vaccine Preparations

**Figure 3:**
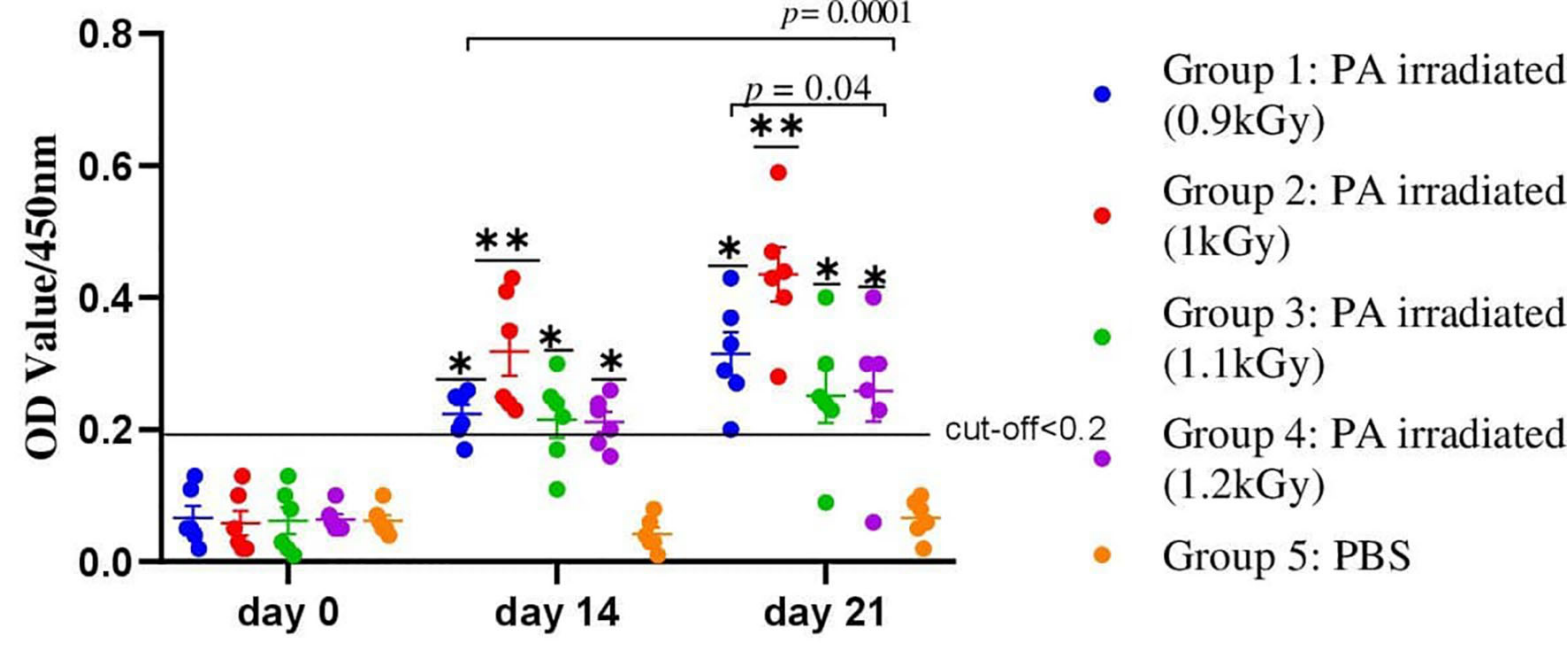
A total of 30 chickens were assigned into five groups, with each group having six chickens. Chickens in groups 1, 2, 3, and 4 received 5.6×10^9^ CFU/ml of avian *P. multocida* irradiated with 0.9, 1, 1.1, and 1.2 kGy of gamma rays and adjuvanted with Montanide/01 PR gel, respectively, through IN route. Chickens in group 5 received PBS and were used as a control. Blood samples were taken at days 0, 14, and 21. Serum was analyzed for the presence of IgG. The data were presented as individual values for each experimental group. Mean and standard error of means are indicated in lines and error bars. Asterisk (*) represents the significant differentiation of antibody IgG level compared to the non-vaccinated control group (*p < 0.05, **p < 0.01).

The safety and immunogenicity of several vaccine preparations made from avian *P. multocida* treated with various doses of gamma radiation adjuvanted with Montanide/01 PR gel (SEPPIC, France) was investigated. Briefly, 30 chickens were assigned into five groups (six chickens per group); four groups were administered 5.6×10^9^ CFU/ml of avian *P. multocida* irradiated with 0.9, 1, 1.1, and 1.2 kGy of gamma ray formulated with Montanide/01 PR gel through IN route. Another group of six chickens received PBS and was used as a control. At 14 and 21 days after vaccination, significantly higher levels of PA-specific IgG antibodies were identified in the serum of the four vaccinated groups when compared to the PBS-inoculated control groups (**Figure 3**). In chickens immunized with irradiated avian *P. multocida* at 1 kGy, the average antibody titer was 0.345 ± 0.095 on day 14 and 0.43 ± 0.12 on day 21, while in chickens immunized with 0.9 kGy irradiated was 0.22 ± 0.06 and 0.31 ± 0.11. On the other hand, chickens immunized with 1.1 and 1.2 kGy irradiated avian *P. multocida* produced similar average antibody titer 0.21 ± 0.06 on day 14 and 0.26 ± 0.11 on day 21. When compared to the other vaccinated chickens (different gamma irradiation doses), chickens vaccinated with 1 kGy gamma irradiation candidate vaccine generated significantly higher levels of IgG antibodies in the serum at days 14 and 21 post-vaccination (*p*=0.0001). Furthermore, chickens vaccinated with 0.9 kGy gamma-irradiated candidate vaccine had higher levels of antibodies as compared to the remaining groups (*p*=0.04). There was no clinical evidence of sickness or injection site reactions in any of the vaccinated chickens. In all vaccinated groups, no lesion was found in the liver or spleen of chickens. Irradiation at 1 kGy was chosen as optimum irradiation dose based on immunogenicity and safety data.

### Evaluation of Serum and Mucosal Antibody Response Against Mucosal FC Vaccine

**Figure 4:**
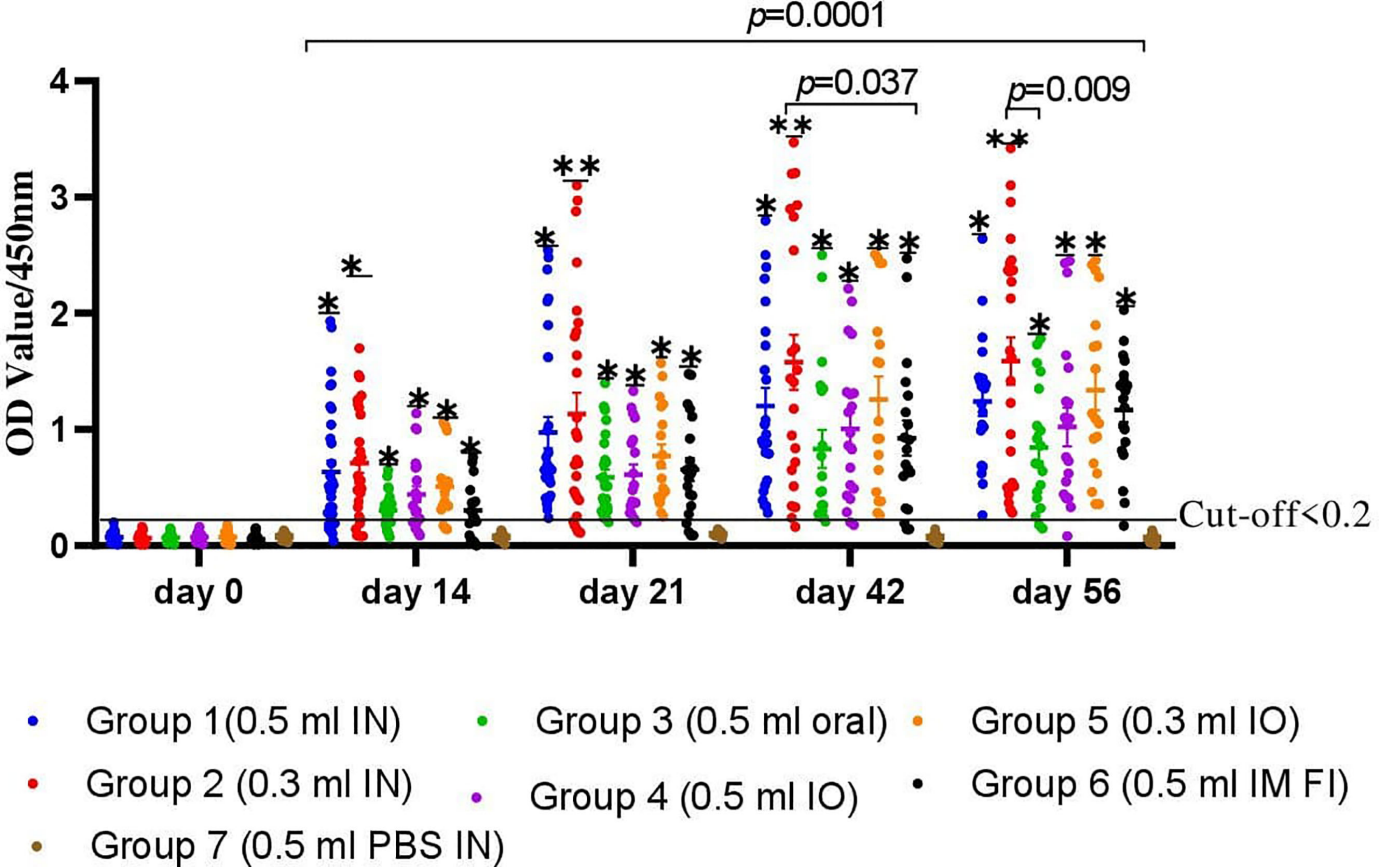
A total of 220 chickens were assigned into seven groups: G-1 to G-5 based on the dose of candidate gamma-irradiated FC vaccine they received and the route of administration. Chickens in both G-1 and G-2 received the vaccine by intranasal route at a dose of 0.5 and 0.3 ml, respectively. Similarly, chickens in G-3 received the vaccine at a dose of 0.5 ml orally. On the other hand, G-4 and G-5 were administered 0.5 and 0.3 ml of the vaccine through the intraocular route (IO), respectively. Another two groups of chickens, G-6 and G-7, received 0.5 ml of the commercial formalin-inactivated FC vaccine intramuscularly and 0.3 ml of PBS intranasally as comparator, respectively. Blood samples were taken at days 0, 14, 21, 42, and 56. Serum was analyzed for the presence of IgG. The data were presented as individual values for each experimental group. Mean and standard error of means are indicated in lines and error bars. Asterisk (*) represents the significant differentiation of antibody IgG level compared to the non-vaccinated control group (*p < 0.05, **p < 0.01).

Determination of serum IgG titers using an indirect ELISA is shown in **Figure 4**. The levels of antibody titers of chickens against FC on day 0 indicated a low cutoff value of 0.2. The levels of chicken serum IgG titers were found to be significantly increased after 2 weeks of the first immunization with the gamma-irradiated or formalin-inactivated vaccine. At the third week, a significant difference was observed among all vaccinated groups, where the average antibody titer of G-2 was 1.13 ± 0.16 compared to 0.97 ± 0.18, 0.59 ± 0.096, 0.61 ± 0.08, 0.77 ± 0.17, and 0.65 ± 0.15 for G-1, G-3, G-4, G-5, and G-6, respectively. After the second vaccination, the titers substantially increased in all chickens and were still significantly higher in the vaccinated chickens of gamma-irradiated mucosal FC vaccine than in the corresponding formalin-inactivated vaccinated group (*p*=0.037) (**Figure 4**). Furthermore, peak of average antibody titer in G-1, G-2, and G-5 was observed after the second vaccination, which is 1.2 ± 0.18, 1.58 ± 0.29, and 1.26 ± 0.24, respectively. As compared to the other gamma-irradiated FC vaccinated groups, the average antibody titer of group-3 (0.83 ± 0,23) was low and showed significant difference compared to Group-2 (*p*<0.009). However, there was no significant difference between the mean average antibody titer of groups of chickens vaccinated orally with the irradiated FC vaccine (G-3) and those vaccinated with commercial formalin-inactivated FC vaccine (G-6) throughout the experiment. After the third vaccination dose, the results of mean antibody titer of chickens in all groups were similar with second vaccination dose. On the other hand, the non-immunized group was found to be seronegative to FC, as the average antibody levels throughout the experimental period was lower than the cutoff value. Generally, the results indicated that the gamma-irradiated vaccine formulations are able to induce serum IgG against avian *P. multocida*. With regard to the route of vaccine administration, the average antibody titer levels of group 2 (0.3 ml, intranasal route) generated significantly higher levels of serum IgG throughout the experiment (*p*=0.001). In addition, group-5 (0.3 ml, intraocular route) produced significantly higher antibody (IgG) titer after booster immunization (*p*=0.001).

**Figure 5:**
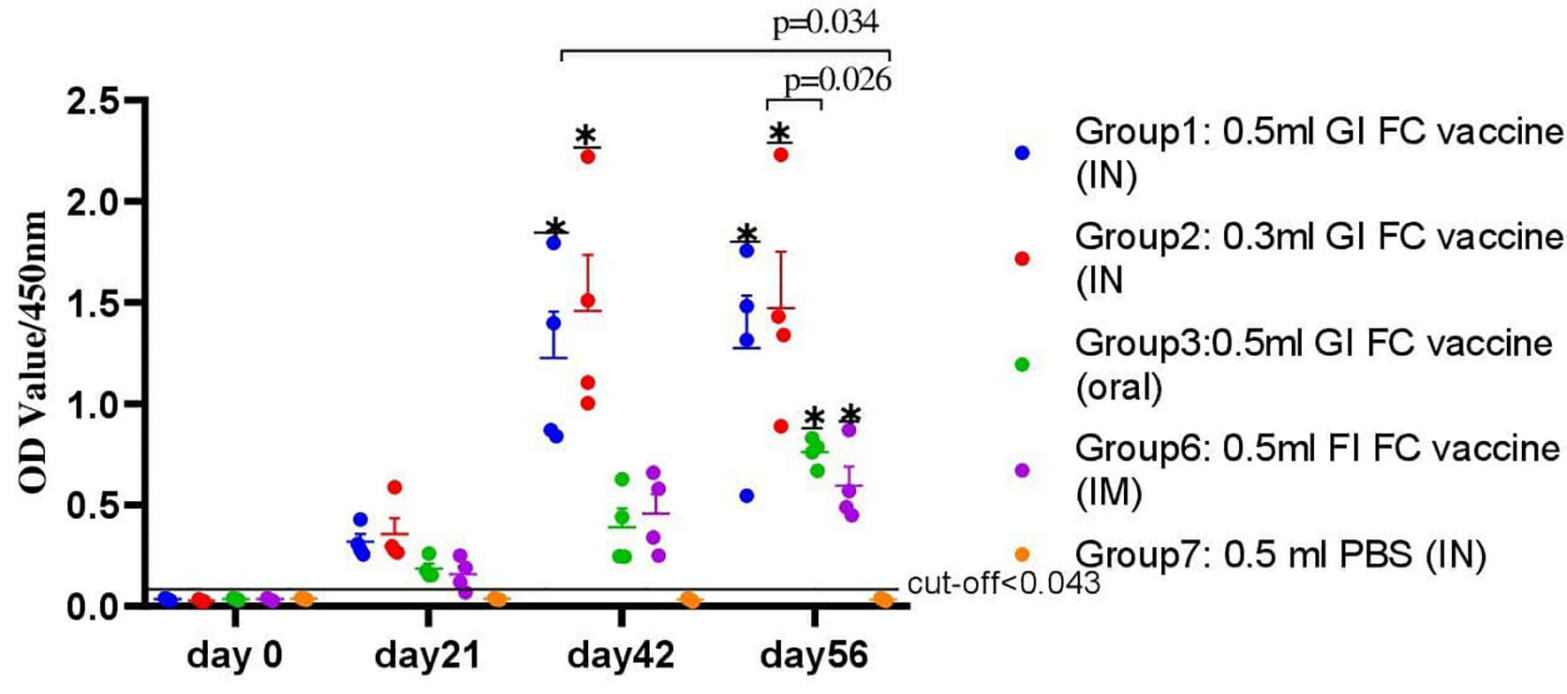
A total of 80 chickens were chosen from all groups. Tracheal (G-1, G-2, G-4, and G-7) and crop lavage (G-3) was taken from four chickens from each group that were euthanized on different period of interval: day 0, 21, 42, and 56 of the experiment; thus, a total of 16 chickens from each group were euthanized for this purpose. Trachea and crop were washed by glycine buffer and analyzed for the presence of IgA. The data were presented as individual values for each experimental group. Mean and standard error of means are indicated in lines and error bars. Asterisk (*) represents the significant differentiation of antibody IgA level compared to the non-vaccinated control group (*p < 0.05).

Secretory IgA was also detected in chickens immunized with the candidate gamma-irradiated mucosal fowl cholera vaccine (**Figure 5**). The levels of average IgA titers of chickens against FC on day 0 indicated a low cutoff value of 0.043. At the third week, a significant difference was observed among chickens vaccinated with the gamma-irradiated vaccine intranasally at two dose rates, where the average IgA titer of G-1 and G-2 was 0.32 ± 0.05 and 0.36 ± 0.12 compared to the control group (0.036 ± 0.02). After the second vaccination, the titers substantially increased in all chickens. Comparative evaluation with formalin-inactivated FC vaccine showed that chickens vaccinated with the gamma-irradiated candidate vaccine displayed significantly higher average antibody titer of 1.23 ± 0.06 and 1.46 ± 0.22 in G-1 and G-2, respectively, than the chickens immunized with formalin-inactivated vaccine with 0.46 ± 0.09 mean value. After the third vaccination dose, a significant difference was observed among all vaccinated groups compared to the control group (*p*<0.05). Like that of the serum IgG of chickens, the average IgA titers of the chickens vaccinated with gamma-irradiated FC vaccine orally were low and significantly different compared to the intranasal route (*p*=0.026). In the control groups, no response was observed in antibody titers to avian *P. multocida* throughout the experiment. Generally, significant levels of IgA were detected only in gamma-irradiated mucosal fowl cholera immunized chickens, but not in that of formalin-inactivated fowl cholera immunized chickens (*p*=0.034). These results suggest that gamma-irradiated mucosal fowl cholera vaccine is more potent in enhancing or inducing avian *P. multocida*–specific antibodies on the airway mucosal surface more than formalin-inactivated fowl cholera vaccine.

### Evaluation of Protective Efficacy of Mucosal FC Vaccine

A total volume 0.5 ml of bacterial suspension containing 3.5×10^9^CFU/ml of avian *P. multocida* biotype A was administered intranasally. Complete protection of the chickens from fowl cholera was conferred by vaccination with the intranasal route of gamma-irradiated fowl cholera vaccine or by intraocular route of gamma-irradiated fowl cholera (0.3 ml/dose) vaccine. The protective efficacy in the chickens immunized with the gamma-irradiated oral vaccine and formalin-inactivated FC vaccine was 85 and 80%, respectively, while in those immunized with the gamma-irradiated intraocular (0.5 ml) vaccine was 90%. According to the log-rank test for equality survival function, vaccinated groups in both vaccine types showed significant difference compared to the control group (p<0.001). Furthermore, there was significant difference between the protection conferred by the gamma-irradiated mucosal fowl cholera vaccine and formalin-inactivated FC vaccine (p<0.001). In addition, the survival rate of chickens vaccinated with gamma-irradiated FC vaccine intranasally was significantly different compared to chickens vaccinated through the oral route (p<0.001).

**Figure 6:**
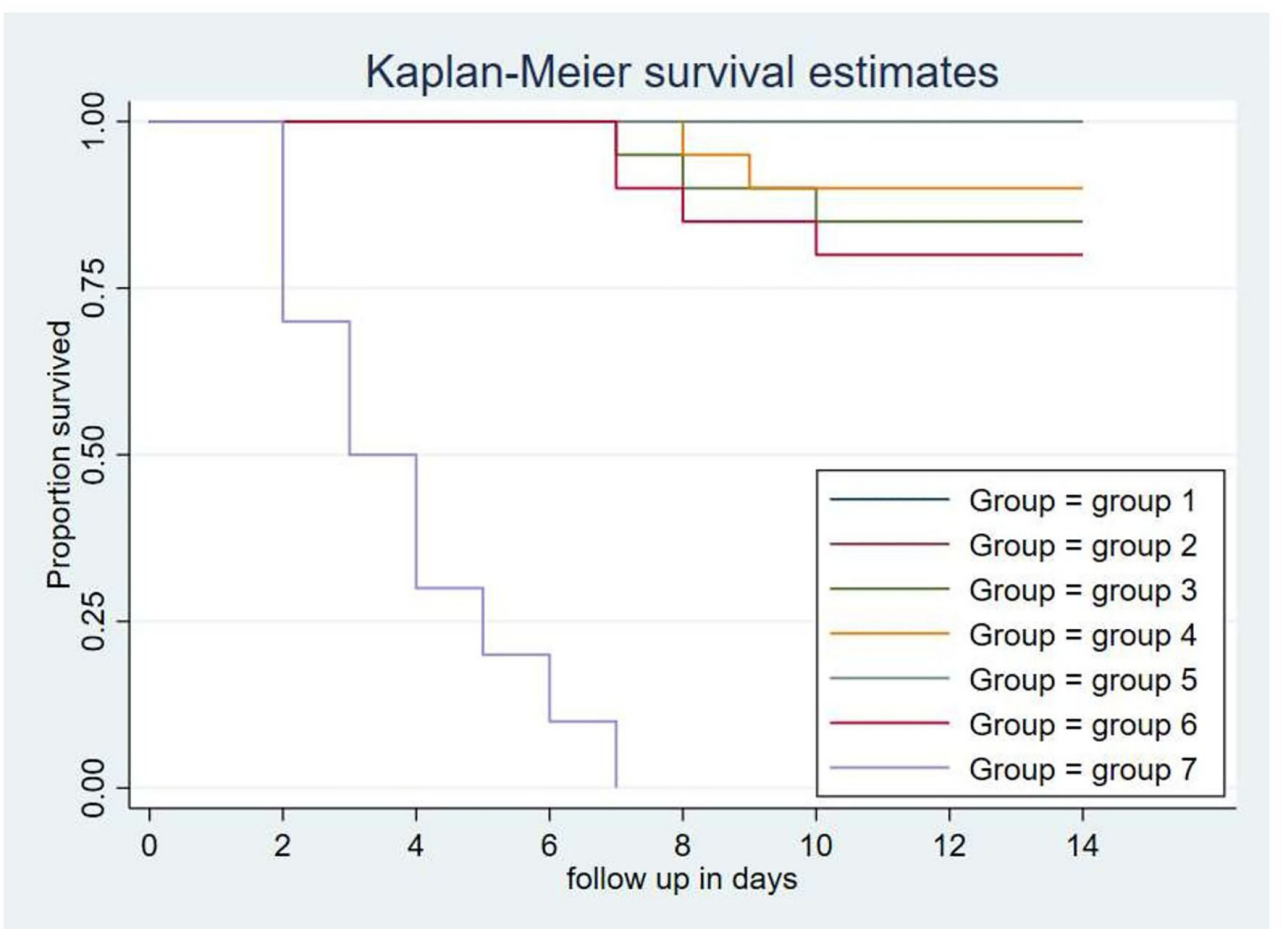
Each group comprises 20 chickens that had been followed for the period of 14 days. The chickens in the treatment and control groups were given 0.5 ml of avian *P. multocida* biotype A. The data were used to determine the Kaplan-Meier estimates (the product limit estimate) of both the control and the treatment groups. The curve takes a step down when the chickens were dead.

Based on the curve, there was not any chicken that survived after exposure to the challenge bacterial strain in the control group. The death of chickens started 2 days after challenge, and all of the chickens in this group died within 7 days. In addition, three and four chickens in gamma-irradiated mucosal FC vaccine through oral route and formalin-inactivated immunized groups, respectively, and two chickens in the gamma-irradiated mucosal FC vaccine through intraocular route (0.5 ml/dose) immunized group died within 10 days, respectively (**Figure 6**).

### Clinical Signs, Gross Lesions, and Bacterial Isolation

After challenge, no behavioral changes were detected in the groups of chickens immunized with gamma-irradiated mucosal FC vaccine through intranasal and intraocular (0.3 ml/dose) routes. In the control groups, chickens manifested clinical signs at 24 h after the challenge exposure, including depression, anorexia, and severe diarrhea. During the period of 4 days, the severity increased rapidly, resulting in the death of several chickens, and all of the chickens in this group died within 7 days. In addition, seven and eight chickens in the gamma-irradiated mucosal FC vaccine through oral route and formalin-inactivated immunized groups, respectively, and five chickens in the gamma-irradiated mucosal FC vaccine through intraocular route (0.5 ml/dose) immunized group started to display depression and anorexia 5 days after the challenge exposure. Among these chicken, three, four, and two chickens then died within 10 days, respectively.

**Figure 7:**
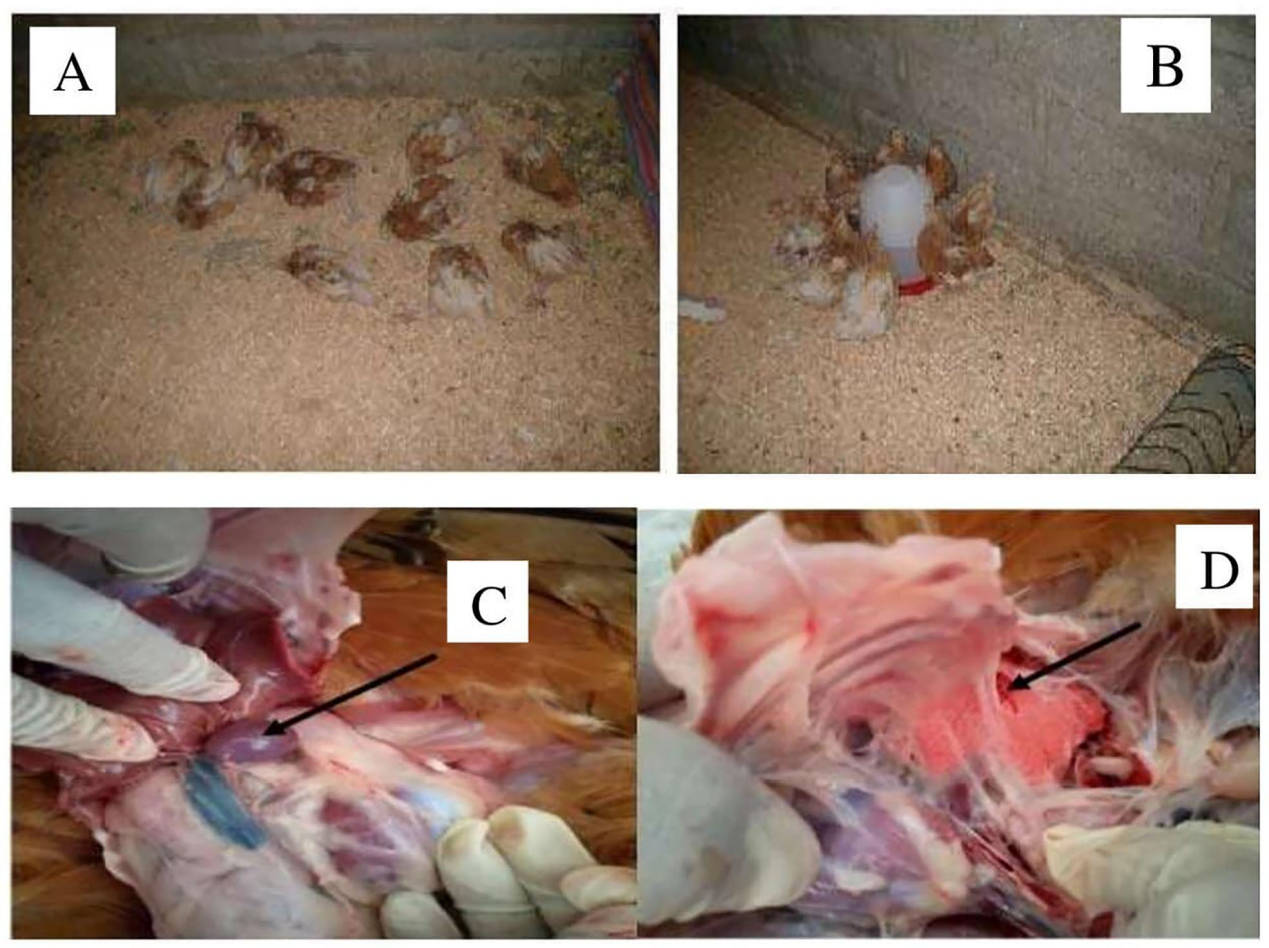
The picture from **(A–D)** indicated the chickens died after challenge with *P. multocida* biotype A at day 56 and the postmortem result. **(A, B)** indicated the dead chickens within 2 weeks. **(C, D)** indicated splenomegaly and petechial and congested lung.

All of the dead chickens in this investigation had characteristic lesions of fowl cholera, including lung congestion, lung edema, and numerous petechiae in the liver, hemorrhage in the small intestine, splenomegaly, and fibrinopurulent peritonitis, according to necropsy results (**Figure 7**). In addition, the dead chickens were also subjected to isolation and identification of avian *P. multocida*. The findings revealed that avian *P. multocida* was recovered in pure cultures from all of the dead chicken specimens, which was furthermore confirmed by species-specific PCR.

## Discussion

Fowl cholera caused by *P. multocida* is a highly contagious disease of poultry presenting as one of the major challenges worldwide. It affects the poultry industry, incurring economic losses due to loss of products (19). The development of vaccine to control FC has proven to be a challenge for years. An effective vaccine must be safe and needs to provide sustained protection with elimination of the challenge infection. Live and formalin-inactivated FC vaccines have been extensively used and succeeded in reducing infection and the prevalence of disease in poultry but have limitations associated with safety and elicit protections of short duration (20). In addition, parenteral vaccines have limited ability of inducing mucosal immunity, which is key in protection against infection or disease by mucosal pathogens. Furthermore, parenterally administered vaccines are stressful to birds and not suitable for mass vaccination, requiring much labor and time. Currently, there is a need for mucosal vaccines against pathogens that invade *via* the mucosal surfaces. This route of vaccine delivery would also eliminate needle injections (7).

The present study is aimed to develop and evaluate gamma-irradiated mucosal FC vaccine that can be utilized nationally to curb the impact of the disease in Ethiopia as well as in other African countries. In a preliminary study we conducted, fresh cultures of avian *P. multocida* (5.6×10^9^ CFU/ml) were irradiated with different doses (0.9, 1, 1.1, and 1.2 kGy) of gamma radiation. Then, the safety and immunogenicity of these preparations [after addition of Montanide gel/01 PR adjuvant (18)] were evaluated in chickens to select the superior radiation dose. Accordingly, irradiation with 1 kGy resulted in safe and immunogenic preparation as evidenced by the higher titer of antibody elicited in chickens and its safety.

Gamma irradiation has been used extensively as an alternative inactivation method of pathogens because of its high penetrability, which allows bacterial inactivation in large volumes within a short time *via* damage of nucleic acid (11). Several previous reports have suggested that nucleic acids rather than proteins are the primary targets of gamma irradiation to inactivate microorganisms. For example, gamma-irradiated *S. pneumoniae* vaccine produced after inactivation with 10 kGy gamma irradiation elicits strong mucosal and systemic immune responses in mice model, which is indicative of the affectivity of gamma irradiation as a method for the development of a killed whole-cell pneumococcal vaccine (21). Furthermore, sterilization doses for radiation-sensitive organisms could be significantly reduced, which would be expected to reduce damage to epitopes required to develop a protective immune response while maintaining an adequate margin of safety to ensure complete inactivation (22).

In our study, a dose range of 1–3 kGy completely inactivated the avian *P. multocida* as confirmed by the subculturing on TSA and TSB while retaining its immunological properties. There are no available reports on inactivation of avian *P. multocida* using gamma rays. However, *M. haemolytica* was reported to be successfully irradiated using gamma rays of 20 kGy doses, a dose selected to be optimal for vaccine preparation (23). According to our study, radiation with 1 kGy resulted in no avian *P. multocida* cell survival, and the resultant vaccine formulation induced significantly higher antibody response than the formalin-inactivated vaccine. This implies that gamma irradiation efficiently inactivates bacteria with less impact on antigenic structures (determinants), leading to a robust immune response. In contrast, formalin inactivation has been known to induce the formation of methylene bridges between amino groups, resulting in protein cross-linking, affecting antigenicity (24).

One of the key determinants of effectiveness of vaccines is the adjuvant selection. Montanide gel/01 PR is an innovative polymeric adjuvant designed to improve the safety and efficacy of aqueous vaccines. Those adjuvants are based on a dispersion of highly stable gel particles of sodium polyacrylate in water (25). The depot effect with slow release due to polymer adsorption properties improves the recruitment of the innate immune system. It provides a significant enhancement of systemic and mucosal immune responses with a better safety performance than potassium aluminum sulfate (Alum) (26). Based on our finding, it can be speculated that the presence of Montanide gel in our vaccine formulation contributed for its better immunogenicity and efficacy as compared to formalin-inactivated FC vaccine. However, this requires further investigation.

Avian *P. multocida* is known to cause disease in poultry species by infecting or entering through the mucosal surface of the upper respiratory tract. Thus, the first line of defense of the host is invoked against inhaled antigens, making the respiratory route potentially the most effective route for vaccination that is capable of inducing both systemic and mucosal immunity (8). Mucosal vaccines administered through IN route mimics the route of natural infection of mucosal pathogens such as avian *P. multocida*, which in turn would result in protective immune response than injectable preparations (6).

The gamma-irradiated mucosal FC vaccine developed in this study was evaluated for its ability to induce both serum IgG and mucosal IgA in chickens. In chicken sera, IgG is the most common immunoglobulin form, and secretory IgA is produced locally by plasma cells located at mucosal surfaces and plays an important role in mucosal immunity (27). This finding indicated that antibody titers in sera of chickens vaccinated with gamma-irradiated mucosal vaccine were significantly increased after 2 weeks post-vaccination, but significant shooting was recorded at 6 and 8 weeks post-vaccination and is in agreement with (8) and (28), who stated that rOmpH-LTB-based intranasal and irradiated bacterial vaccines generate higher humoral immune responses and protection against extracellular and intracellular bacteria and (29) who registered that vaccines developed by irradiation have been found to be strong inducers for humoral immune responses that make this type of vaccine highly effective.

An interesting finding in our study was the gamma-irradiated mucosal fowl cholera vaccine led to high levels of *P. multocida*–specific serum IgG responses as compared to formalin-inactivated fowl cholera vaccine. This might be due to highly preserved immunogenic properties of protein antigens even after irradiation. However, formalin inactivation can cause crosslinking of several amino acid residues, which leads to a lower immunogenic response (30).

In regard to efficacy, our finding showed that vaccination with intranasal and intraocular (0.3 ml/dose) route of the gamma-irradiated mucosal vaccine resulted in 100% protection against lethal challenge. As compared to the intranasal route, immunization through oral and intraocular route resulted in less efficacy (*p*<0.05) as shown by death of some birds in those groups. This can be due to the local IgA produced in the mucosal airways, which is the natural route and of infection of FC (21).

In this study, the gamma-irradiated fowl cholera vaccine was safe as chickens injected with it were devoid of vaccination side effects and their bodies were maintained normal, and it avoids the drawbacks in vaccinated chickens with chemical inactivated fowl cholera vaccines. We believe that these preliminary findings demonstrate that the gamma-irradiated mucosal fowl cholera vaccine approach is an adaptable vaccine strategy against avian *P. multocida* and that this information will aid in the evaluation of other whole-cell, killed vaccine strategies, as well as identify candidates for recombinant protein vaccine approaches.

In conclusion, the present study showed that gamma-irradiated FC mucosal vaccine is safe and protective, suggesting its potential use for immunization of chicken against FC in chicken. One kGy was identified as a dosage of gamma irradiation that inactivated *P. multocida* replication while retaining immunogenic surface structures. Montanide gel/01 PR showed a significant enhancement of systemic and mucosal immune responses with a safety. In this study, the candidate gamma-irradiated mucosal vaccines induced higher response of both serum IgG and mucosal IgA after three IN doses, the latter (IgA) being highly relevant in the context of protective immunity. In addition to its good immunogenicity, the candidate vaccine provided protection in challenge experiments. This can be considered a go-on signal to further evaluate the vaccine and approve it for national use.

## Data Availability Statement

The original contributions presented in the study are included in the article/supplementary material. Further inquiries can be directed to the corresponding author.

## Ethics Statement

The animal study was reviewed and approved by the animal research ethics committee of the National Veterinary Institute, Ethiopia with the protocol number NVI-AE-008-2020.

## Author Contributions

BD and EK were involved in the data collection. BD, DA, and SI participated in the study design and in the laboratory analysis, performed data analysis, and drafted the manuscript. MB, EG, TA, VW, and HU assisted in study conception and manuscript revision and also assisted in analysis interpretation and gave inputs in the final manuscript. EG, TA, VW, and HU read and commented on the manuscript and rearranged for publication. All authors contributed to the article and approved the submitted version.

## Funding

This work was supported by the CRP grant (code D32035) from the International Atomic Energy Agency (IAEA), Vienna, Austria.

## Conflict of Interest

The authors declare that the research was conducted in the absence of any commercial or financial relationships that could be construed as a potential conflict of interest.

## Publisher’s Note

All claims expressed in this article are solely those of the authors and do not necessarily represent those of their affiliated organizations, or those of the publisher, the editors and the reviewers. Any product that may be evaluated in this article, or claim that may be made by its manufacturer, is not guaranteed or endorsed by the publisher.
